# Isthmic Spondylolisthesis in Young Gymnasts: A Report of Two Cases

**DOI:** 10.7759/cureus.35152

**Published:** 2023-02-18

**Authors:** Andreia Silva, Marcel Sincari, Irina Peixoto, Mário Vaz, Eduardo Mendes

**Affiliations:** 1 Physical Medicine and Rehabilitation Department, Tondela-Viseu Hospital Center, Viseu, PRT; 2 Neurosurgery Department, Tondela-Viseu Hospital Center, Viseu, PRT; 3 Orthopaedics and Traumatology Department, Tondela-Viseu Hospital Center, Viseu, PRT

**Keywords:** rehabilitation, orthopedic procedures, spondylolisthesis, neurogenic bladder, sciatica

## Abstract

Isthmic spondylolisthesis is a condition characterized by the subluxation of one vertebral body over the adjacent inferior vertebral body, secondary to a defect in the pars interarticularis. This paper reports two cases of young acrobatic gymnasts who presented with long-term, progressive lumbosciatic pain and lower limb dysesthesia. One gymnast presented with additional neurogenic bladder dysfunction. Diagnostic testing revealed L5-S1 spondylolisthesis due to bilateral isthmic lysis in both gymnasts. Both underwent L4-S2 fixation followed by L5-S1 transdiscal arthrodesis with Rialto sacroiliac fusion system (Medtronic^®^, Minneapolis, MN, USA). Favorable evolution of neuromotor symptoms was observed after rehabilitation, with less residual neurological impairment in the case of higher-grade spondylolisthesis. This case series aims to emphasize the importance of recognizing this pathology in athletes as well as its correct management. Early surgical intervention can prevent the onset and progression of neurological deficits.

## Introduction

Spondylolisthesis results from an anterior, posterior, or lateral translation of a vertebral body relative to its caudal vertebral body [1]. Isthmic spondylolisthesis (IS) corresponds to an injury of the pars interarticularis (PI) which can occur from stress fractures (type IIA), elongation (type IIB), or acute fractures (type IIC) [2-4]. The result is a progressive anterior slippage, most commonly at the L5-S1 vertebral level [1,2].

The degree of the subluxation is categorized according to Meyerding’s classification (grade I: <25%; grade II: 25-50%; grade III: 50-75%; grade IV: 75-100%; grade V: >100%). This scale allows the distinction between low-grade spondylolisthesis (grades I and II), high-grade spondylolisthesis (grades III and IV), and spondyloptosis (grade V) [5]. The Wiltse classification is based on etiology and anatomical factors and may be divided into I - dysplastic; II - isthmic; III - degenerative; IV - traumatic; V - pathological; and VI - iatrogenic [4].

Clinical presentation includes lower back pain with or without radicular symptoms and, rarely, bladder and/or bowel dysfunction [1-3]. There is no clear consensus regarding management, with treatment options ranging from conservative measures for asymptomatic patients or those with mild symptoms to surgical treatment in the face of neurological compromise [2]. Spondylolysis and IS can cause significant morbidity in adolescent gymnasts, occurring in approximately 11%, with repeated trauma and hyperextension of the lumbar spine as the likely injury mechanism [1,3,6,7]. This paper describes two cases of sports-related IS at L5-S1 with partial recovery of neurological deficits.

## Case presentation

Case one

**Figure 1:**
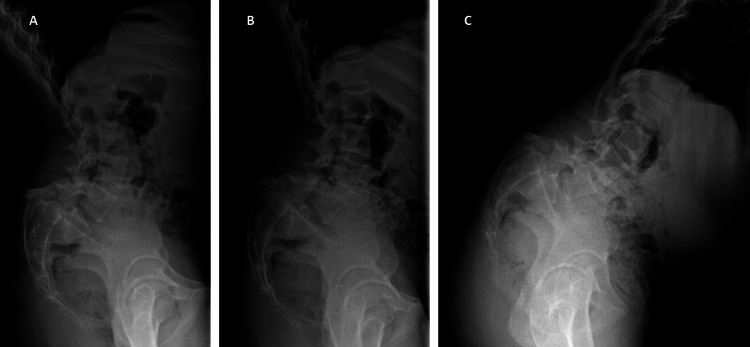
Dynamic radiography with evidence of lumbar hyperlordosis and spondyloptosis in case one. A: Hyperextension. B: Neutral position. C: Hyperflexion.

**Figure 2:**
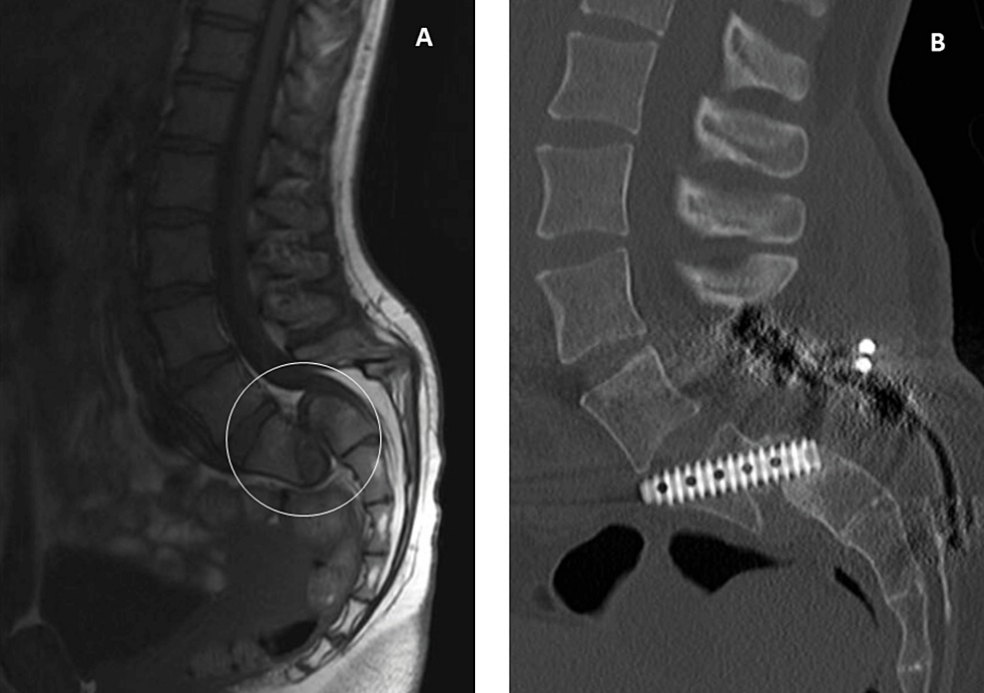
Case one. A: Magnetic resonance imaging of the lumbosacral spine showing grade V spondylolisthesis (circle). B: Postoperative control computed tomography with an illustration of the Rialto sacroiliac arthrodesis system (Medtronic®, Minneapolis, MN, USA).

**Figure 3:**
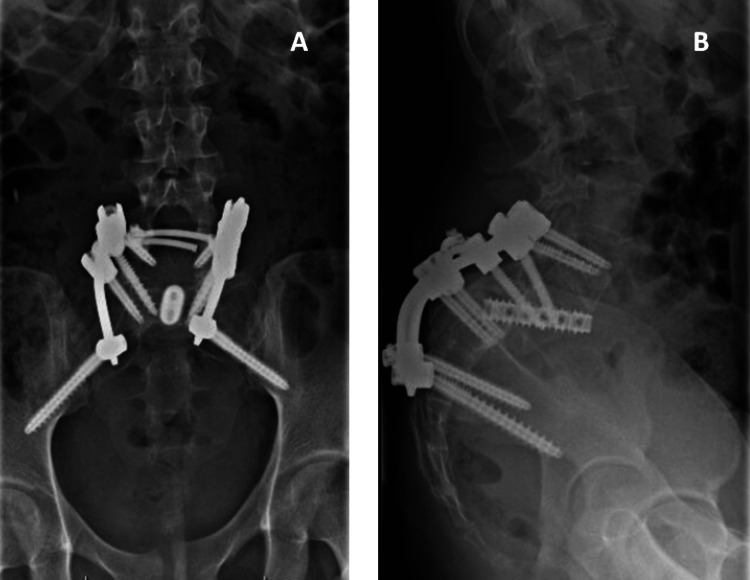
Postoperative control lumbosacral radiographs in case one. A: Anteroposterior view. B: Lateral view.

An 18-year-old female acrobatic gymnast presented with chronic low back pain radiating to the lower limbs (asymmetrical radiation with left predominance) and dysesthesia in the L5 territory for four years. She had normal osteotendinous reflexes, absence of motor deficits, and positive Lasègue’s sign on the left lower limb. Dynamic radiography (DR) revealed grade V spondylolisthesis at L5-S1 and the presence of instability (Figure 1). Magnetic resonance imaging (MRI) showed grade V anterolisthesis at L5 with bilateral isthmic lysis (Figure 2). Electroneuromyography confirmed radiculopathy of the left L5 nerve root (Appendices). The patient underwent surgical intervention using a posterior approach for fixation of L4-S2 with transpedicular screws, L5 laminectomy, insertion of reduction screws, L5-S1 discectomy, and placement of an autologous bone graft. The Rialto sacroiliac fusion system (Medtronic®, Minneapolis, MN, USA) [8] was used to promote L5-S1 fusion (Figures 2, 3). The patient subsequently showed significant improvement in self-reported pain; however, she maintained hypoesthesia in the territory of L5, S1, and S2 on the left lower limb. Pregabalin (150 mg per day) was introduced to manage dysesthesia, and discontinuation of acrobatic gymnastics was recommended.

Case two

**Figure 4:**
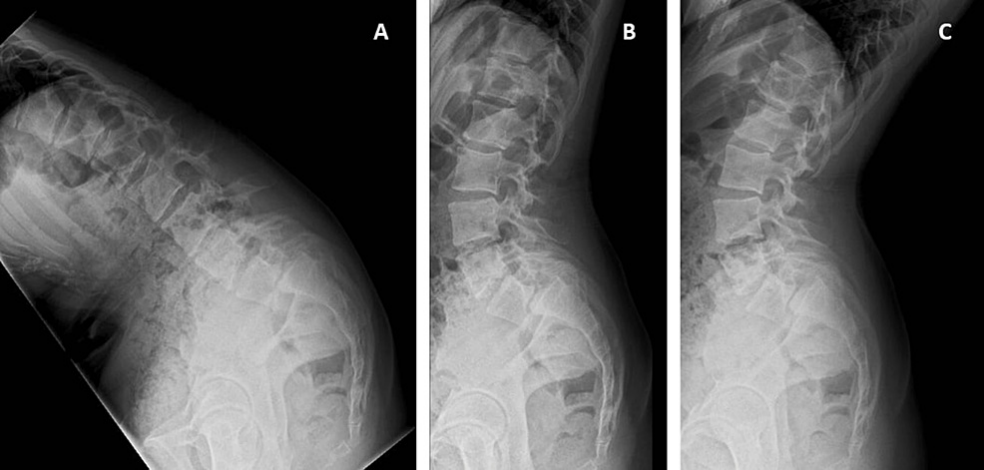
Dynamic radiography with accentuation of lumbar curvature and grade III spondylolisthesis in case two. A: Hyperflexion. B: Neutral position. C: Hyperextension.

**Figure 5:**
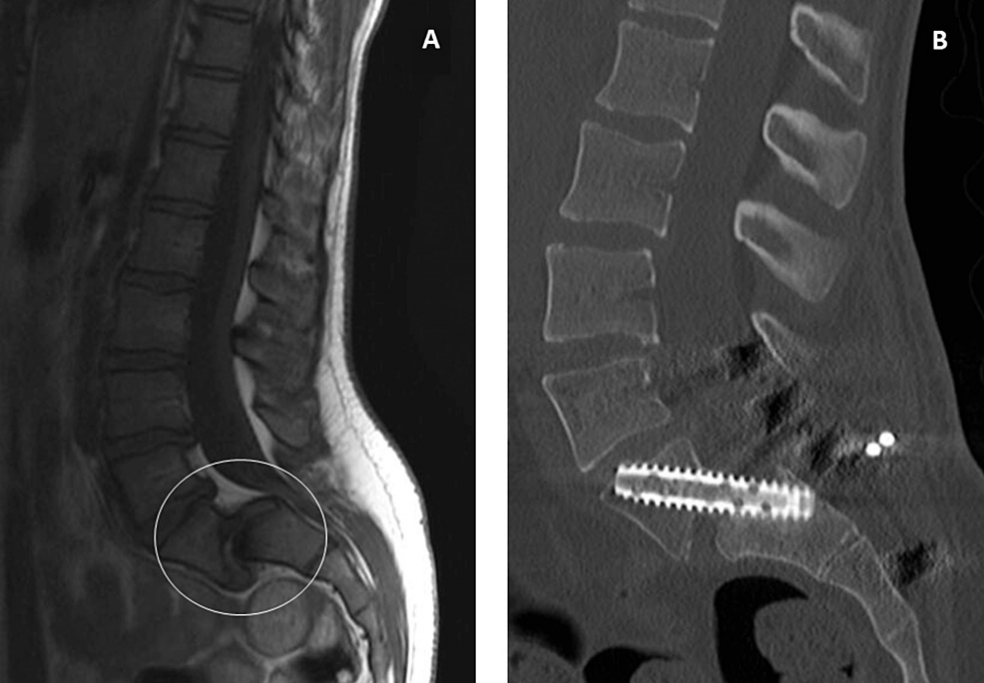
Case 2. A: Magnetic resonance imaging of the lumbosacral spine showing grade III spondylolisthesis (circle). B: Postoperative control computed tomography with an illustration of the Rialto sacroiliac arthrodesis system ((Medtronic®, Minneapolis, MN, USA)).

**Figure 6:**
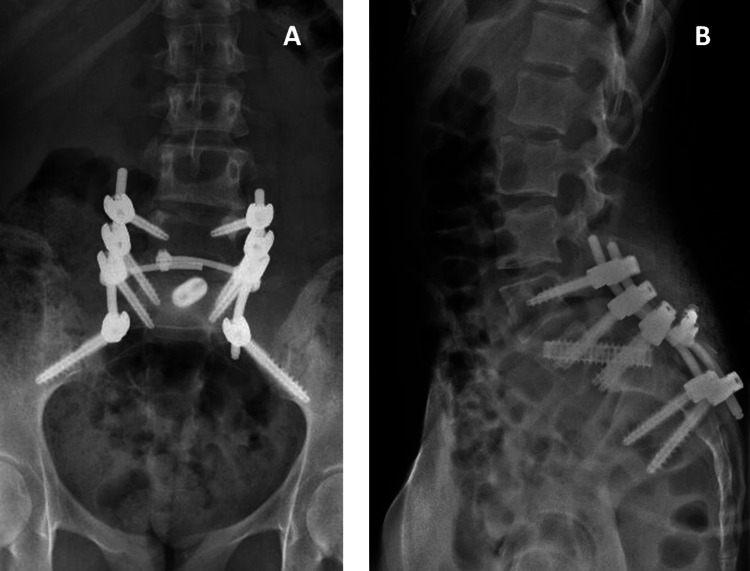
Postoperative control lumbosacral radiographs in case two. A: Anteroposterior view. B: Lateral view.

A 19-year-old female acrobatic gymnast presented with left lumbosciatic pain associated with dysesthesia in L5 and S1 territories as well as urinary urgency episodes for the past three years. She presented a positive Lasègue’s sign on the left lower limb and sensory deficits in the corresponding regions. DR (Figure 4) and MRI (Figure 5) showed grade III spondylolisthesis at L5-S1 due to bilateral isthmic lysis, compromise of the S1 roots, and an occult spina bifida. Electroneuromyography confirmed radiculopathy of the left S1 root with signs of active denervation. The urodynamic study evidenced a neurogenic bladder with the presence of uninhibited detrusor contraction; however, it demonstrated preserved bladder sensitivity and capacity (Appendices). Transpedicular instrumentation of L4-S1 was subsequently performed, with the placement of bilateral sacroiliac screws at S2, L5 laminectomy, and L5-S1 transdiscal arthrodesis with Rialto cylinder (Medtronic®) [8]. As with the first case, the correct positioning of the osteosynthesis material was verified on imaging examination (Figures 3, 6). The patient maintained hypoesthesia in the left gluteal region, and a rehabilitation program with home exercises and suspension of sports practice was recommended.

## Discussion

IS affects 4-8% of the general population [9,10], with a higher prevalence in gymnasts [7,11], especially female athletes (11%) [11,12]. It is usually found at the L5-S1 vertebral level due to the instability of the PI, as described in the presented cases [1]. A defect in this structure leads to anterior subluxation and, consequently, stress fractures [1,4].

The most common clinical symptom is mechanical lower back pain [1-3,10]. Acute IS may also present with impaired bowel and/or bladder function, in addition to motor/sensory deficits of the lower limbs, as observed in case two. On the other hand, in chronic IS, radicular symptoms are more frequent, which is consistent with the clinical presentation of case one [3].

Diagnosis is generally performed with DR, allowing for assessment of spondylolisthesis severity, but MRI is the gold standard in patients with neurological symptoms, possibly requiring surgical intervention [2]. The decision for surgical intervention depends on the severity and persistence of symptoms, neurological impairment, degree of subluxation, inherent instability, and impact on function [2]. In both athletes, the pain was affecting their ability to walk, limiting their practice of gymnastics. Despite the lower degree of spondylolisthesis in case two, her bladder function was also compromised.

Several surgical techniques are available for the treatment of spondylolisthesis, but there is still no consensus regarding which is most effective [3,13]. In the above cases, in addition to L4-S2 fixation and L5 laminectomy, the cylindrical Rialto device (Medtronic®) was used to promote L5-S1 arthrodesis (Figure 6). Although this material was initially designed to facilitate sacroiliac joint arthrodesis, it has already demonstrated its utility in the surgical treatment of this pathology. The authors found two cases of grade III spondylolisthesis reported in the literature in which this arthrodesis system was adopted [14]. The results were positive in terms of symptoms, sagittal balance, deformity correction, and complication rate [14].

Curiously, the patient with greater neurological impairment (radicular symptoms and neurogenic bladder) had a less severe anatomical lesion (grade III vs. grade V). Both patients participated in a rehabilitation program integrating physiotherapy and hydrotherapy, with significant improvement in pain and sensory deficits. They performed daily physiotherapy for four weeks, which consisted of neurosensory and proprioceptive stimulation of the lower limbs, strengthening of the dorsolumbar muscles, postural re-education exercises, and desensitization massage of the surgical scar. In the following three weeks, they started hydrotherapy every two days. It included water gait training with progressive loads for strengthening the core and lower limb muscles, proprioceptive training, and effort reconditioning.

## Conclusions

Sports-related IS can result in serious clinical sequelae. This case series intends to highlight the importance of early diagnosis in the management of IS in gymnasts. Early intervention may prevent the onset and progression of neurological deficits, as well as promote functional recovery. Additionally, further evidence is required to determine the best approach for the management of IS, and the novel surgical technique used in this case series, using the Rialto system to promote healing, should be investigated as an alternative surgical strategy.

## Figures and Tables

**Figure 7:**
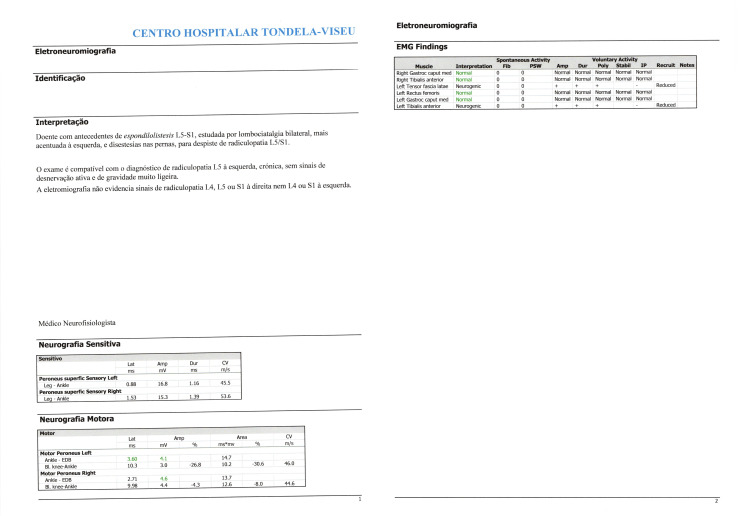
Electroneuromyography in case one.

**Figure 8:**
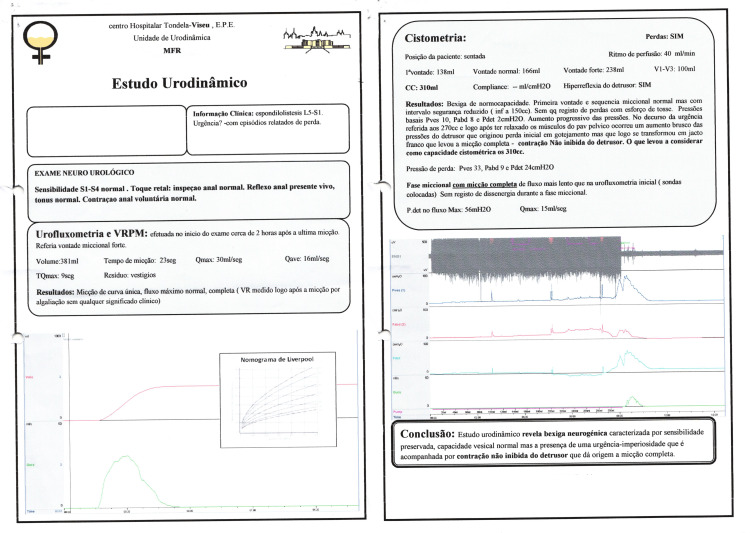
Urodynamic study in case two.

## References

[1] Beck AW, Simpson AK (2019). High-grade lumbar spondylolisthesis. Neurosurg Clin N Am.

[2] Kasliwal MK, Smith JS, Kanter A, Chen CJ, Mummaneni PV, Hart RA, Shaffrey CI (2013). Management of high-grade spondylolisthesis. Neurosurg Clin N Am.

[3] Cavalier R, Herman MJ, Cheung EV, Pizzutillo PD (2006). Spondylolysis and spondylolisthesis in children and adolescents: I. Diagnosis, natural history, and nonsurgical management. J Am Acad Orthop Surg.

[4] Wiltse LL, Newman PH, Macnab I (1976). Classification of spondylolisis and spondylolisthesis. Clin Orthop Relat Res.

[5] Koslosky E, Gendelberg D (2020). Classification in brief: the Meyerding classification system of spondylolisthesis. Clin Orthop Relat Res.

[6] Congeni J, McCulloch J, Swanson K (1997). Lumbar spondylolysis. A study of natural progression in athletes. Am J Sports Med.

[7] Toueg CW, Mac-Thiong JM, Grimard G, Parent S, Poitras B, Labelle H (2010). Prevalence of spondylolisthesis in a population of gymnasts. Stud Health Technol Inform.

[8] (2022). Medtronic. https://www.medtronic.com/us-en/index.html.

[9] Ganju A (2002). Isthmic spondylolisthesis. Neurosurg Focus.

[10] Standaert CJ, Herring SA (2000). Spondylolysis: a critical review. Br J Sports Med.

[11] Crawford CH 3rd, Ledonio CG, Bess RS (2015). Current evidence regarding the etiology, prevalence, natural history, and prognosis of pediatric lumbar spondylolysis: a report from the Scoliosis Research Society Evidence-Based Medicine Committee. Spine Deform.

[12] Jackson DW, Wiltse LL, Cirincoine RJ (1976). Spondylolysis in the female gymnast. Clin Orthop Relat Res.

[13] Rivollier M, Marlier B, Kleiber JC, Eap C, Litre CF (2020). Surgical treatment of high-grade spondylolisthesis: technique and results. J Orthop.

[14] Sharma M, Aljuboori Z, Clouse JW, Rodgers R, Altstadt T (2019). Sacro-iliac joint fusion system for high-grade spondylolisthesis using "Reverse Bohlman technique": a technical report and overview of the literature. World Neurosurg.

